# Factors associated with bypassing primary healthcare facilities for childbirth among women in Devchuli municipality of Nepal

**DOI:** 10.1371/journal.pone.0302372

**Published:** 2024-04-18

**Authors:** Manisha Maharjan, Sudim Sharma, Hari Prasad Kaphle

**Affiliations:** 1 School of Health and Allied Sciences, Pokhara University, Lekhnath, Nepal; 2 Faculty of Public Health, Mahidol University, Salaya, Thailand; Group for Technical Assistance / Asian College for Advance Studies, Purbanchal University, NEPAL

## Abstract

**Background:**

It is crucial to deliver a child at nearby primary healthcare facilities to prevent subsequent maternal or neonatal complications. In low-resource settings, such as Nepal, it is customary to forgo the neighboring primary healthcare facilities for child delivery. Reports are scanty about the extent and reasons for bypassing local health centers in Nepal. This study sought to determine the prevalence and contributing factors among women bypassing primary healthcare facilities for childbirth.

**Method:**

A community-based cross-sectional study was carried out in the Devchuli municipality of Nawalparasi East district of Nepal. Utilizing an online data collection tool, structured interviews were conducted among 314 mothers having a child who is less than one year of age.

**Results:**

This study showed that 58.9% of the respondents chose to bypass their nearest primary healthcare facility to deliver their babies in secondary or tertiary hospitals. Respondent’s husband’s employment status; informal employment (AOR: 4.2; 95% CI: 1.8–10.2) and formal employment (AOR: 3.2; 95% CI: 1.5–6.8), wealth quintile (AOR: 3.7; 95% CI: 1.7–7.7), parity (AOR): 3.0; 95% CI: 1.6–5.7], distance to nearest primary healthcare facility by the usual mode of transportation (AOR: 3.0; 95% CI: 1.5–5.6) and perceived service quality of primary healthcare facility (AOR: 3.759; 95% CI: 2.0–7.0) were associated with greater likelihood of bypassing primary healthcare facility.

**Conclusion:**

Enhancing the quality of care, and informing beneficiaries about the importance of delivering children at primary healthcare facilities are essential for improving maternal service utilization at local primary healthcare facilities.

## Introduction

Access to quality maternal healthcare services is crucial for ensuring positive maternal and neonatal outcomes [1]. In many developing countries, including Nepal, the healthcare system is designed with a hierarchical framework to facilitate effective patient referral, starting from primary level birthing centers and escalating to higher-level facilities when necessary [2]. This referral system aims to optimize healthcare service utilization, allocate resources efficiently, and provide specialized and timely care to those in need [3].

Despite the availability of emergency obstetric care services at primary health facilities in Nepal, there has been a notable trend of pregnant women bypassing these facilities and directly seeking childbirth services at tertiary-level hospitals without referral [4,5]. It is reported that among the 17 higher-level hospitals offering comprehensive emergency obstetric and neonatal care (CEONC), 12 experienced overcrowding, exceeding the capacity of available beds. Consequently, women seeking care were compelled to use makeshift beds on the floor [6]. As a result, the bypassing practice raises concerns about the underutilization of primary healthcare resources and the overcrowding of higher-level facilities [2]. Consequently, this phenomenon results in an increased burden on tertiary hospitals, leading to potential risks of infection, medical errors, and dissatisfaction among patients, while also escalating healthcare expenses for individuals and families [5,7].

Understanding the reasons behind bypassing primary health facilities for childbirth is crucial for developing effective strategies to strengthen the healthcare delivery system and enhance maternal and neonatal health outcomes. Previous studies have identified various factors influencing the decision to bypass, such as perceptions of low quality of healthcare at primary health facilities, availability of specialized services at higher-level facilities, community awareness of the referral system, and readiness of the nearest facility to provide childbirth services [4,8,9]. Despite the recognition of bypassing as a significant issue, research on the proportion and determinants of bypassing primary health facilities for childbirth remains limited in Nepal. As such, it is crucial to closely understand the status and determinants of bypassing primary healthcare for childbirth.

By comprehending individual preferences and perceptions regarding healthcare facilities for childbirth, this study can contribute to guiding appropriate policy interventions in optimizing healthcare utilization, enhancing maternal and neonatal outcomes and promoting equitable access and improving maternal healthcare services at the primary level [10]. The primary objective of this study was to assess the status and factors associated with bypassing primary healthcare facilities for childbirth among women in Devchuli Municipality.

## Materials and methods

### Study design and study setting

A cross-sectional quantitative study design was used to assess the status and factors associated with bypassing the primary healthcare (PHC) facilities for childbirth. The study was conducted in Devchuli Municipality of Nawalpasari East District, Nepal consisting of 17 sub-division wards. The municipality consists of 8840 households [11]. There are six basic health service centers (BHSC), three health posts and a primary healthcare center (PHCC). There is a private hospital in the municipality, however, the delivery service is not available in the private hospital. While the municipality consists of two birthing centers. A health post provides birthing services and a PHCC providing Basic Emergency Obstetric and Neonatal care (BEONC) services. The data was collected between 25 June and 25 July 2023.

### Study population and sampling procedure

Women who had given birth during the previous year were randomly selected for this study. Women who delivered a baby within the last 12 months in health institutions were included in the study. Whereas women who delivered a baby other than health institutions and those who couldn’t communicate due to their physical or mental conditions were excluded from this study.

The sample size was based on Cochran [12] and taking the preceding prevalence of 55% into account, the margin of error was set to 0.05%, and 95% as a confidence interval, a total sample size (n) of 380 was calculated. Later adjusting the sample size for a finite population with a total of 1096 expected live births; at Devchuli municipality in the fiscal year 2022/23 [11] and adding a 10% non-response rate, the desired sample size was 314. The sampling frame was created by compiling data obtained from both the municipality and health facilities. After its preparation, the total study population was determined to be 598 individuals. Using a simple random sampling technique, data was collected from 314 respondents, randomly selected through computer-generated random numbers derived from the sampling frame. The Female Community Health Volunteers (FCHVs) played a key role in locating the respondents within the community.

### Study variables

There are 12 socio-demographic variables which include, the age of the respondents, ethnicity, religion, family type, education of the respondents, respondents’ husband’s education, occupation of the respondents, respondents’ husband’s occupation, wealth index, enrollment to health insurance, women’s autonomy in decision making for obstetric care and presence of co-morbidity during last pregnancy. The obstetric variables include 4 variables: gravida, parity, number of Antenatal Care (ANC) visits and previous experience of obstetric complication. Similarly, the health facilities variables include the type of nearest health facility, distance to the nearest health facility, perceived service quality of PHC facility and perceived competencies of health workers of PHC facility. The status of bypassing PHC facilities is measured by 3 questions regarding place of delivery, referred from primary healthcare facilities and presence of obstetric complications in the last pregnancy. Place of delivery is recorded in terms of Health post/ Birthing Center, Primary Health Care Center, Government Hospital and Private Hospital/ Clinics. Similarly, referred from primary healthcare facilities and presence of obstetric complication is measured in terms of ‘yes’ and ‘no’. The women who delivered their last child in the government hospital and private hospital/ clinics without having pre-identified complications and were not referred from the primary healthcare facilities are termed as bypassing primary healthcare facilities.

### Operational definition

The dependent variable, bypassing primary healthcare facilities, refers to women who delivered their last child in secondary or tertiary health facilities without pre-identified complications and without being referred by primary healthcare (PHC) facilities, including birthing centers and Basic Emergency Obstetric and Newborn Care (BEONC) centers. It is coded as 1 for bypassing and 0 for no bypassing.

Primary healthcare facility refers to the primary level health facility which includes a public health facility with birthing services and BEONC services.

Family type refers to the structure of the family where the respondent lives. It is attributed into nuclear, joint, and extended.

Wealth index refers to the ownership of the selected assets by the households which was measured by the 12 questions of International Wealth Index (IWI) and IWI score was calculated in the total of 100 and classified into five groups with the interval of 20 score [13].

Perceived service quality of primary healthcare facility refers to the perception of the women about the service quality of the primary healthcare facilities. The quality was measured in terms of nine dimensions using the Likert scale (Disagree; Neutral; Agree), a) adequate human resources, b) laboratory services, c) medicine supplies, d) equipment supplies, e) clean water and electricity, f) adequate private room, g) ambulance service, h) opening time and i) waiting time. Each statement was given the score. The level of perception was classified based on the median score where less than median score is termed as poor perception and more than median score is termed as good perception.

Perceived competences of health workers are the perception of the women about the competences of the health workers working in the primary health facilities. The competences of the health workers were measured in terms of four dimensions using the Likert scale (Disagree; Neutral; Agree), a) examination duration, b) behavior towards patients, c) handling birth related complications and d) counseling and providing information. The level of perception was classified based on the median score where less than median score is termed as poor perception and more than median score is termed as good perception.

### Tools and techniques of data collection

Data was collected through a face-to-face interviewer-administered questionnaire. The data was collected in ODK Collect in tablet/ mobile. The consistency and completeness of the data were checked after the completion of the data collection.

The questionnaires in this study were based on a thorough literature review. The tool was initially developed in English and later translated into the local Nepali language. For content validity, we consulted experts having similar experience. The tool was pre-tested among 32 participants in the neighboring municipality and later adjusted and modified before the data collection. For internal consistency reliability test was performed for perception-related Likert scale items by using Cronbach’s alpha where it was reported at 0.732 for perceived service quality-related statements and 0.769 for perceived competencies of health workers-related statements, which was greater than the recommended value of 0.70.

### Data processing and analysis

The data was collected and stored in the Open Data Kit (ODK) platform and was reviewed and checked for inconsistencies. We secured the anonymized data in a password protected ODK account that is only accessible to the principal investigator and study team. The stored data was later transferred to SPSS (Statistical Package for Social Science) version 26 for further analysis. Frequency, mean, and standard deviation were used for descriptive statistics. The Chi-square test, Bi-variate and multiple logistic regression analysis were performed.

### Ethical considerations

The Ethical approval from the Institutional Review Committee (IRC) of Pokhara University was obtained (reference number 150-079/080). Written approval to conduct the study was also acquired from Devchuli municipality. Written informed consent was taken from each of the participants before the interview and confidentiality of the participants was maintained.

## Results

The study’s outcomes are structured into three parts. The initial section offers contextual information. The subsequent section outlines how often women bypass PHC facilities for childbirth, with figures presented as frequency, percentage, mean, standard deviation, and median. The final section identifies the factors linked to bypassing PHC facilities, using multiple logistic regression. Table 1 presents the socio-demographics of the women: the majority group (41.1%) was aged 25–29, with a mean age of 26.04 and a standard deviation of 4.424 years. Most respondents (95.5%) identified as Hindus, while the majority (50.6%) were Janajati. About 66.2% belonged to joint/extended families. Likewise, 74.8% had secondary education, and 76.1% of husbands also attended secondary school. Homemakers/students accounted for 86% of respondents, while 46.2% of husbands had formal jobs. Nearly half (43.9%) fell in the highest wealth quintile. About 64% of respondents held decision-making autonomy in healthcare. Co-morbidity during the last pregnancy was seen in 4.1% of women. Community involvement was observed in over two-thirds (64.6%) of respondents.

**Table 1:** Socio-demographic characteristics of women.

Characteristics	Frequency	Percent
**Age of respondents**		
Less than 20	22	7.0
20–24	94	29.9
25–29	129	41.1
30–34	55	17.5
35 and above	14	4.5
Mean ± SD: 26.04± 4.424		
**Religion**		
Hindu	300	95.5
Christian	8	2.5
Muslim	6	1.9
**Ethnicity**		
Brahmin/Chhetri	102	32.5
Janajati	159	50.6
Dalit	47	15.0
Muslim	6	1.9
**Family type**		
Nuclear	106	33.8
Joint/Extended	208	66.2
**Respondents’s educational status**		
Non-formal education	14	4.5
Basic education	40	12.7
Secondary education	235	74.8
Graduate or above	25	8.0
**Husband’s educational status**		
Non-formal education	4	1.3
Basic education	48	15.3
Secondary level	239	76.1
Graduate or above	23	7.3
**Respondent’s occupation**		
Homemaker/ students	282	89.8
Informal employemnt	14	4.5
Formal employement	18	5.7
**Husband’s occupation**		
Unemployed/ students	60	19.1
Informal employemnt	109	34.7
Formal employement	145	46.2
**Wealth quintile**		
Poorest	0	0.0
Second	15	4.8
Middle	68	21.7
Fourth	93	29.6
Highest	138	43.9
**Health insurance enrollment**		
No	237	75.5
Yes	77	24.5
**Women’s autonomy in decision making in obstetric care**		
No	113	36.0
Yes	201	64.0
**Involved in community activities**		
No	111	35.4
Yes	203	64.6
**Presence of comorbidity**		
No	301	95.9
Yes	13	4.1
**Gravida**		
Primigravida	137	43.6
Multigravida	177	56.4
**Parity**		
Primiparous	139	44.3
Multiparous	175	55.7
**Number of ANC visits**		
< 4 ANC	9	2.9
≥ 4 ANC	305	97.1
**Experienced obstetric complications in previous pregnancy (n = 177)**		
No	159	89.8
Yes	18	10.2
**Mode of transportation**		
Walking	41	13.1
Private vehicle	43	13.7
Public vehicle	230	73.2

Over half of the women had multigravida (56.4%) and 55.7% had multiparous. Additionally, nearly all women (97.1%) had undergone four or more ANC visits during their most recent pregnancies. Out of 277 respondents, 10.2% encountered obstetric complications in previous pregnancies. Regarding delivery, the majority (72.9%) gave birth in government hospitals, while 16.9% chose primary health care centers; a small portion (1.9%) delivered in health posts or birthing centers. Similarly, 73.2% utilized public transportation for ANC visits, while 13.1% opted to walk.

Table 2 shows health facilities-related information. Over half (52.9%) of the respondents traveled 31–60 minutes for delivery services at the health facility, and 28.3% traveled over 60 minutes. Similarly, most respondents (77.7%) took 15 or more minutes to reach the nearest primary healthcare facility using their usual mode of transport. Among all respondents, nearly two-thirds (63.1%) considered the service quality of the primary healthcare facility as good, while more than two-thirds (70.7%) perceived the competencies of health workers at the primary healthcare facility as good.

**Table 2:** Health system-related characteristics.

Characteristics	Frequency	Percent
**Distance to health facility where delivered**		
≤ 30 minutes	59	18.8
31–60 minutes	166	52.9
> 60 minutes	89	28.3
**Distance to the nearest primary healthcare facility by usual means of transportation**		
< 15 mins	70	22.3
≥ 15 mins	244	77.7
**Perceived service quality of primary healthcare facility**		
Poor	116	36.9
Good	198	63.1
**Perceived competencies of health workers of primary healthcare facility**		
Poor	92	29.3
Good	222	70.7

Table 3 shows the status of bypassing primary healthcare facilities. Regarding the place of delivery, most respondents (72.9%) gave birth at government hospitals, followed by BEONC centers (16.9%). Obstetric complications were encountered by 21.7% of women in their last pregnancy. Likewise, 16.5% were referred by PHC facilities. More than half (58.9%) bypassed the PHC facility, opting for secondary or tertiary hospitals for childbirth.

**Table 3:** Status of bypassing primary healthcare facility.

Characteristics	Frequency	Percent
**Place of delivery**		
Health post with birthing center	6	1.9
Primary healthcare center/ BEONC	53	16.9
Government hospital	229	72.9
Private hospital	26	8.3
**Experienced obstetric complications in last pregnancy**		
No	246	78.3
Yes	68	21.7
**Referred by primary healthcare facility**		
No	213	83.5
Yes	42	16.5
**Bypassed primary healthcare facility**		
No	129	41.1
Yes	185	58.9

### Factors associated with bypassing the primary level healthcare facility for childbirth

The result shows the association of socio-demographic characteristics with bypassing the primary level healthcare facility for childbirth. Categories of variables such as religion, ethnicity, education status and wealth quintile were merged to perform further analysis due to the presence of fewer than five expected observations in each cell. Table 4 shows that ethnicity, educational status the education of the respondents’ husband, the occupation of the respondents’ husband and wealth quintile and co-medical illness of the respondents were found to be statistically significant with p-value < 0.05.

**Table 4:** Association of sociodemographic characteristics with bypassing primary healthcare facility.

Characteristics	Bypassing primary healthcare facility		Chi square value	df	p-value
	Yes	No			
	(n; %) (185; 58.9)	(n; %) (129; 41.1)			
**Age of the respondent**					
Less than 20	9 (40.9)	13 (59.1)	7.272	4	0.122
20–24	60 (63.8)	34 (36.2)			
25–29	79 (61.2)	50 (38.8)			
30–34	27 (49.1)	28 (50.9)			
35 and above	10 (71.4)	4 (28.6)			
**Religion**					
Hindu	177 (59.0)	123 (41.0)	0.019	1	0.890
Non- Hindu	8 (57.1)	6 (42.9)			
**Ethnicity**					
Brahmin/Chhetri	70 (68.6)	32 (31.4)	5.885	1	0.020*
Non- Brahmin/Chhetri	115 (54.2)	97 (45.8)			
**Type of family**					
Nuclear	69 (65.1)	37 (34.9)	2.523	1	0.112
Joint/Extended	116 (55.8)	92 (44.2)			
**Education of respondent**					
Up to basic education	21 (38.9)	33 (61.1)	10.808	1	0.001*
Secondary education or above	164 (63.1)	96 (36.9)			
**Husband’s education**					
Up to basic education	23 (44.2)	29 (55.8)	5.553	1	0.018*
Secondary education or above	162 (61.8)	100 (38.2)			
**Occupation of respondent**					
Homemaker/ students	167 (59.2)	115 (40.8)	1.949	2	0.377
Informal occupation	6 (42.9)	8 (57.1)			
Formal occupation	12 (66.7)	6 (33.3)			
**Husband’s occupation**					
Unemployed	28 (46.7)	32 (53.3)	9.255	2	0.010*
Informal occupation	59 (54.1)	50 (45.9)			
Formal occupation	98 (67.6)	47 (32.4)			
**Wealth quintile**					
Middle or less	31 (37.3)	52 (62.7)	21.682	1	<0.001*
Higher	154 (66.7)	77 (33.3			
**Health insurance enrollment**					
No	146 (61.6)	91 (38.4)	2.881	1	0.090
Yes	39 (50.6)	38 (49.4)			
**Women’s autonomy in decision making in obstetric care**					
No	65 (57.5)	48 (42.5)	0.142	1	0.706
Yes	120 (59.7)	81 (40.3)			
**Presence of co-morbidity**					
No	184 (61.1)	117 (38.9)	14.702^**a**^	1	<0.001*
Yes	1 (7.7)	12 (92.3)			
**Women’s involvement in community activities**					
No	67 (60.4)	44 (39.6)	0.148	1	0.701
Yes	118 (58.1)	85 (41.9)			

*Statistically significant at the level of p-value <0.05.

^a^ Fisher exact test.

The Table 5 shows the association of obstetric-related factors of the women with bypassing the primary level healthcare facility for childbirth. The result shows that parity, complications experienced in previous, and last pregnancies, mode of transportation and ever visited nearest health facility were found to be statistically significant with p-value < 0.05.

**Table 5:** Association of obstetric-related factors with bypassing primary healthcare facility.

Characteristics	Bypassing primary healthcare facility		Chi square value	df	p-value
	Yes	No			
	(n; %) (185; 58.9)	(n; %) (129; 41.1)			
**Gravida**					
Primigravida	89 (65.0)	48 (35.0)	3.671	1	0.055
Multigravida	96 (54.2)	81 (45.8)			
**Parity**					
Primiparous	91 (65.5)	48 (34.5)	4.421	1	0.035*
Multiparous	94 (53.7)	81 (46.3)			
**No. of ANC**					
< 4 ANC	5 (55.6)	4 (44.4)	0.043^**a**^	1	1.000
≥ 4 ANC	180 (59)	125 (41)			
**Experienced obstetric complications in previous pregnancy (n = 177)**					
No	80 (50.3)	79 (49.7)	9.694^**a**^	1	0.002*
Yes	16 (88.9)	2 (11.1)			
**Experienced obstetric complications in last pregnancy**					
No	185 (75.8)	61 (24.2)	124.476^**a**^	1	<0.001*
Yes	0 (0.0)	68 (100.0)			
**Mode of transportation to reach the health facility**					
Walking	16 (39.0)	25 (61.0)	8.356	2	0.015*
Own vehicle	29 (67.4)	14 (32.6)			
Public vehicle	140 (60.9)	90 (39.1)			
**Nearest health facility**					
Health post/ Birthing center	28 (68.3)	13 (31.7)	0.399	1	0.528
Primary health care center/ BEONC	155 (73.1)	57 (26.9)			

*Statistically significant at the level of p-value <0.05.

^a^ Fisher exact test.

The Table 6 shows the association of health system-related factors with bypassing the primary level healthcare facility for childbirth. The result shows that all the health system-related variables that is time to travel to the health facility for delivery services, Distance to the nearest the primary healthcare facility by usual means of transportation, perceived quality of health facility and perceived competences of health workers were found to be statistically significant with p-value < 0.05 in Pearson’s chi-square test.

**Table 6:** Association of health system-related factors with bypassing primary healthcare facility.

Characteristics	Bypassing primary healthcare facility		Chi square value	df	p-value
	Yes	No			
	(n; %) (185; 58.9)	(n; %) (129; 41.1)			
**Distance to the health facility where delivered**					
≤ 30 minutes	0 (0.0)	59 (100.0)	104.189^a^	1	<0.001*
> 30 minutes	185 (72.5)	70 (27.5)			
**Distance to the nearest the primary healthcare facility by usual means of transportation**					
< 15 mins	29 (41.2)	41 (58.6)	11.383	1	0.001*
≥ 15 mins	156 (63.9)	88 (36.1)			
**Perceived service quality of primary healthcare facility**					
Poor	88 (75.9)	28 (824.1)	21.822	1	<0.001*
Good	97 (49.0)	101 (51.0)			
**Perceived competencies of health workers of primary healthcare facility**					
Poor	65 (70.7)	27 (29.3)	7.403	1	0.007*
Good	120 (54.1)	102 (45.9)			

*Statistically significant at the level of p-value <0.05.

Table 7 presents the outcomes of logistic regression analysis, highlighting variables with significant links to bypassing PHC facilities for childbirth. Variables that demonstrated significance in Pearson’s chi-square test (p-value < 0.05) were included in the logistic regression analysis. The multicollinearity test was performed to assess correlations among independent variables. This was achieved using the variance inflation factor (VIF) and the tolerance test, which did not reveal multicollinearity issues. The unadjusted odds ratio and adjusted odds ratio were calculated for each independent variable that showed significant association in the chi-square test, utilizing the enter method. The result indicates that the respondents whose husbands have informal employment are four times more likely to bypass the primary healthcare facility (AOR: 4.3; 95% CI: 1.8–10.2) and the respondents whose husbands have formal employment are thrice more likely to bypass the primary healthcare facility (AOR: 3.2; 95% CI: 1.5–6.9) compared to unemployed. Regarding the wealth quintile, the respondents who had the higher wealth are almost four times more likely to bypass the primary healthcare facility (AOR: 3.7; 95% CI: 1.7–7.7) as compared to the respondents with the middle of below wealth.

**Table 7:** Logistic regression analysis of factors associated with bypassing the primary level healthcare facility for childbirth.

Variable	Bypassing primary health facilities for delivery				
	UOR	95% CI	AOR	95% CI	p-value
**Ethnicity**					
Non-Brahmin/Chhetri (ref)	1		1		
Brahmin/Chhetri	1.8	1.1–3.0	1.7	0.9–3.3	0.124
**Education**	** **				
Basic education or below (ref)	1		1		
Secondary education or above	2.7	1.5–4.9	1.6	0.5–5.2	0.412
**Husband’s employment**					
Unemployed (ref)	1		1		
Informal employment	1.3	0.7–2.5	3.3	1.3–8.2	0.009*
Formal employment	2.4	1.3–4.4	2.6	1.2–5.8	0.015*
**Wealth quintile**					
Middle or below (ref)	1		1		
Higher	3.4	2.0–5.7	3.8	1.8–8.1	0.001*
**Parity**					
Multiparous (ref)	1		1		
Primiparous	1.6	1.0–2.6	3.0	1.6–5.7	<0.001*
**Mode of Transportation**					
Walking (ref)	1		1		
Private vehicle	3.2	1.3–7.9	1.6	0.6–4.8	0.375
Public vehicle	2.4	1.2–4.8	1.7	0.8–4.0	0.185
**Distance to nearest primary healthcare facility by usual means of transportation**					
< 15 mins (ref)	1		1		
≥ 15 mins	2.5	1.5–4.3	3.0	1.5–5.6	0.001*
**Perceived service quality of primary healthcare facilities**					
Good (ref)	1		1		
Poor	3.272	2.0–5.4	3.7	2.0–7.0	<0.001*
**Perceived competences of health workers of primary healthcare facilities**					
Good (ref)	1		1		
Poor	2.046	1.2–3.4	1.3	0.7–2.6	0.430

*Statistically significant at the level of p-value < 0.05.

AOR = Adjusted odds ratio.

CI = Confidence Interval.

UOR = Unadjusted odds ratio.

ref = Reference category.

Similarly, the respondents with primiparous status are three times more likely to bypass primary healthcare facilities (AOR: 3.0; 95% CI: 1.6–5.7) compared to women with multiparous status. The odds of bypassing the PHC facility are three times higher among the respondents whose nearest primary healthcare facilities are at a distance of 15 minutes or more (AOR: 3.0; 95% CI: 1.5–5.6). Similarly, the respondents having a poor perception regarding the service quality of the nearest PHC facility are almost four times more likely to bypass PHC facilities (AOR: 3.8; 95% CI: 2.0–7.0) as compared to the respondents with a good perception regarding the quality of nearest PHC. Whereas the adjusted odds ratio does not show a significant result between the respondents having poor perception regarding the competencies of the health workers of the nearest health facility and bypassing the PHC facility.

## Discussion

### Status of bypassing primary healthcare facility

We assessed the status and the associated factors of bypassing primary healthcare for childbirth. Results show that, despite the increasing popularity of institutional delivery in Nepal, there has been a simultaneous surge in bypassing primary healthcare facilities. This study reveals that around 58.9% of women opted for secondary or tertiary hospitals for childbirth, bypassing their nearest primary healthcare facility, even without complications or referral. This figure is slightly higher (55%) than a similar study in Chitwan, Nepal by Shah in 2016 [14]. However, it’s lower than Kaski, Nepal, where 70.2% of women who delivered in health facilities bypassed the closest birthing facility [4].

A comparable outcome emerged in Afghanistan, with 59% of women bypassing primary healthcare facilities for childbirth [15]. In contrast, bypassing was less prevalent in Kusulu [16] and Pwani [8] districts of Tanzania, and Eastern Uganda [17], with rates of 42%, 41.8%, and 29% respectively.

Facility-based studies in neighboring Indian states, Gujarat [18] and Madhya Pradesh [19] showed lower rates of bypassing primary health facilities for childbirth compared to our study, at 37.7% and 39% respectively. Conversely, these rates were higher than studies in Ghana (33.3%) [20], South Ethiopia (30.9%) [21], and Marsabit, Kenya (47.29%) [22]. On the other hand, bypassing was significantly higher in Dilla and Dawan administration [6], Eldoret in Kenya [23], and Tanzania [24], with rates of 65.6%, 76.7%, and 75.4% respectively, surpassing our study. These disparities likely stem from varying healthcare systems among these countries, influencing the quality, accessibility, availability, distribution, and types of obstetric care facilities.

### Factors affecting bypassing primary health facility

This study showed that the husband’s occupation has a significant association with bypassing the primary healthcare facility for childbirth. Those with informal employment were four times and those with formal employment were three times more likely to bypass PHC facilities. A similar study in South Ethiopia found a comparable correlation [21]. This indicates that with increasing household income, individuals have an enhanced ability to cover healthcare expenses. As a result, they might opt for traveling greater distances to access more advanced and sophisticated healthcare facilities, rather than opting for the nearest primary healthcare facility. Similarly, wealthier households showed almost fourfold odds of bypassing the PHC facilities for childbirth. This is in line with the findings in Chitwan, Nepal [14], Kaski [4], Nepal, and Uganda [17]. Greater wealth often corresponds with increased healthcare accessibility, encouraging families to seek services from more distant but advanced facilities. Despite the availability of free hospital delivery services and transportation incentives for women in Nepal, considerable expenses can still arise due to additional medication, travel, and accommodation. Traveling farther to seek healthcare services at a higher level in the cities increases the cost of accommodations for those accompanying pregnant women. Hence, financially disadvantaged families are less likely to bypass the nearest PHC facilities, which could lead to extra expenses [4,25].

Similarly, the current study found that primiparous women were three times more likely to bypass primary healthcare facilities for childbirth. This aligns with similar studies in Chitwan [14] and Kaski [4], Nepal, indicating that women with higher parity were less likely to bypass these facilities. In Tanzania, first-time mothers were over twice as likely to bypass primary health facilities [8]. This discrepancy could be attributed to multiparous women’s better familiarity with maternal health services due to prior pregnancies, while primiparous women might have heightened concerns about risk, prompting their utilization of primary healthcare facilities.

The study indicates that the odds of bypassing PHC facilities are nearly three times higher among respondents who’s nearest PHC facilities are located at a distance of 15 minutes or more. The result is in line with other studies that have also observed an increase in the odds of bypassing primary healthcare facilities with an increased distance to the nearest healthcare facility [6–8]. Bypassing the nearest PHC facilities contributes to obstetric complications, leading to fatal outcomes. This risk is particularly significant if the bypass covers a substantial distance, such as moving to the higher-level health facilities in the city areas. Moreover, it can result in a negative community perception about the bypassed facility, leading to reduced utilization of nearby PHC facilities [26,27].

This study revealed that respondents with negative perceptions about the service quality of the nearest primary health facility are nearly four times more likely to bypass it compared to those with positive perceptions. Low-quality perception significantly drives primary healthcare facility bypassing [28,29]. Studies indicate that women are attracted to hospitals due to perceived higher technical quality, as they offer comprehensive emergency maternity care, which primary healthcare facilities do not provide adequately [30,31]. Similar findings in Tanzania show that perceiving poor quality at primary health facilities increases the likelihood of seeking birthing services elsewhere. Additionally, an Ethiopian study supports this by linking bypassing to the absence of medical supplies in nearby primary health facilities [8].

Additional research has similarly discovered a strong correlation between bypassing and the perceived as well as objectively observed quality of care provided at both the bypassed facility and the chosen alternative. Healthcare establishments with limited medical staff, inadequate medication supplies, and substandard infrastructure were more prone to being bypassed. Similar findings were documented in Namibia, where patients who opted for bypassing expressed concerns regarding the quality of health services [30,32]. Furthermore, bypassing creates undue pressure on the chosen facility and indirectly impacts other women seeking obstetric care, contributing to prolonged wait times, delayed emergency care, unequal distribution of skilled attendants, and childbirth-related complications. This avoidance of available childbirth facilities holds serious implications for maternal healthcare service provision and the human resources of a healthcare system [4,20,21,27].

### Strength and limitation

This study marks the inaugural research conducted within study area focusing on assessing the prevalence of bypassing primary healthcare facilities and identifying the factors that contribute to this phenomenon.

One notable limitation of our study is that it focused exclusively on individuals who delivered live births in the past 12 months. We chose to exclude those who experienced pregnancy losses, such as miscarriages and stillbirths, during this period. While this decision was made to streamline the study’s focus, it does limit the generalizability of our findings to the broader population of individuals who have been pregnant in the past year. Future research should consider including a more diverse sample to explore the experiences of those with different pregnancy outcomes. In addition, there is the possibility of recall bias during the data collection process. This study assesses the service user’s perspective on bypassing primary healthcare facilities. However, this study is unable to identify the factors from the health service provider’s perspective.

## Conclusion

This study reveals that 6 out of 10 women bypass primary healthcare facilities for normal childbirth. This study found parity, husband’s occupation, wealth quintile, and perceived service quality as associated factors for bypassing primary healthcare facilities. The study highlights the pivotal role of primary healthcare service quality in determining facility utilization.

To counter the prevailing trend of bypassing primary healthcare facilities, it is recommended to actively advocate for the utilization of these facilities for both normal deliveries and emergency obstetric services. This can be achieved through tailored interventions designed for women and their families, specifically targeting the alleviation of strain on secondary and tertiary health facilities. Moreover, awareness programs should encompass all women, their husbands, and families, regardless of parity or socio-economic status, during antenatal visits. The integration of information highlighting the advantages of utilizing primary healthcare facilities into routine antenatal education sessions is crucial. Diverse communication channels, including community gatherings and social media, should be utilized to ensure a broader reach.

There is an immediate need for the enhancement of the quality of care for both normal deliveries and Basic Emergency Obstetric and Newborn Care (BEONC) services in primary healthcare facilities. This improvement is essential to augment the utilization of childbirth services. The implementation of these targeted interventions, tailored to factors influencing women’s decisions, holds significant promise in mitigating the prevalent practice of bypassing primary healthcare facilities for childbirth.

## Supporting information

S1 Text Questionnaire. (DOCX)
