# Supplementary material for: Factors associated with bypassing primary healthcare facilities for childbirth among women in Devchuli municipality of Nepal
Source: PLoS One. 2024 Apr 18;19(4):e0302372. doi: 10.1371/journal.pone.0302372 (PMC11025753; doi:10.1371/journal.pone.0302372)
Supplement: S1 Text — (DOCX) [file pone.0302372.s001.docx]

**Questionnaire**

**Factors Associated with Bypassing Primary Health Care Facilities for Child Birth Among Women in Devchuli Municipality, Nawalparasi East district, Nepal**

| Form No.: | ID No.: | Date: |
| --- | --- | --- |

| **Q.N.** | **Question** | **Response** | **Code** |
| --- | --- | --- | --- |
| A | **Socio- demographic information:** | |  |
| A1 | What is your completed age? | ……………years |  |
| A2 | Which religion do you follow? | Hinduism | 1 |
|  |  | Buddhist | 2 |
|  |  | Christianity | 3 |
|  |  | Muslim | 4 |
|  |  | Others (Specify)….......... | 5 |
| A3 | What is your ethnicity? | Brahamin/Chhetri | 1 |
|  |  | Janajati | 2 |
|  |  | Muslim | 3 |
|  |  | Dalit | 4 |
|  |  | Others (Specify)….......... | 5 |
| A4 | Type of family | Nuclear | 1 |
|  |  | Joint | 2 |
|  |  | Extended | 3 |
| A5 | What is your level of education? | Illiterate | 1 |
|  |  | Non-formal | 2 |
|  |  | Basic education | 3 |
|  |  | Secondary level | 4 |
|  |  | Graduate or above | 5 |
| A6 | What is your husband’s level of education? | lliterate | 1 |
|  |  | Non-formal | 2 |
|  |  | Basic education | 3 |
|  |  | Secondary level | 4 |
|  |  | Graduate or above | 5 |
| A7 | What is your occupation? | Homemaker | 1 |
|  |  | Agriculture | 2 |
|  |  | Labour | 3 |
|  |  | Business | 4 |
|  |  | Government Services | 5 |
|  |  | Private Services | 6 |
|  |  | Others (Specify)….......... | 7 |
| A8 | What is your husband’s occupation? | Unemployed | 1 |
|  |  | Agriculture | 2 |
|  |  | Labor | 3 |
|  |  | Business | 4 |
|  |  | Government Service | 5 |
|  |  | Private Service | 6 |
|  |  | Foreign Employment | 7 |
|  |  | Others (Specify)….......... | 8 |
| A10 | Who decided the health facility for the delivery of your last child? | Self | 1 |
|  |  | Husband | 2 |
|  |  | In- Laws | 3 |
|  |  | Others (Specify)….......... | 4 |
| A11 | Did you independently decide to spend during your last child birth? | Yes | 1 |
|  |  | No | 2 |
| A12 | Did you had any co-medical illness during the pregnancy of your last child? | Yes | 1 |
|  |  | No | 2 |
| A13 | Is your family enrolled in health insurance? | Yes | 1 |
|  |  | No | 2 |
|  | **Does the household own or have a:** | |  |
| A14 | TV | Yes | 1 |
|  |  | No | 2 |
| A15 | Refrigerator | Yes | 1 |
|  |  | No | 2 |
| A16 | Telephone/ mobile | Yes | 1 |
|  |  | No | 2 |
| A17 | Bike | Yes | 1 |
|  |  | No | 2 |
| A18 | Car | Yes | 1 |
|  |  | No | 2 |
| A19 | Cheap Utensils (< Npr. 5000) (Household owing one or more cheaper assets , like having at least one or more chair, table, clock, watch, water cooker, radio, fan or mixer) | Yes | 1 |
|  |  | No | 2 |
| A20 | Expensive Utensils (> Npr 30000) (Household owing at least one or more items, like having washer, dryer, computer, moterbike, motorboat, air conditioner or generator) | Yes | 1 |
|  |  | No | 2 |
| A21 | Electricity | Yes | 1 |
|  |  | No | 2 |
|  | **What is the quality of the...** |  |  |
| A22 | Main Source of drinking water? | Low quality (Unprotected well, spring or surface water) | 1 |
|  |  | Middle quality (Public tap or protected well) | 2 |
|  |  | High quality (Bottled water or water piped) | 3 |
| A23 | Toilet facility usually used? | Low quality | 1 |
|  |  | (Traditional pit latrine or no toilet facility |  |
|  |  | Middle quality (Public toilet, improved pit latrine) | 2 |
|  |  | High quality (Private flush toilet) | 3 |
| A24 | Main floor material? | Low quality (None, soil, dung) | 1 |
|  |  | Middle quality (Cement, concrete, wood) | 2 |
|  |  | High quality (Finished floor with parquet, carpet, titles, linoneum, ceramic) | 3 |
| A25 | No. of rooms used for sleeping | One | 1 |
|  |  | Two | 2 |
|  |  | Three or more | 3 |

| **B** | **Obstetric Information** | **Response** | **Code** |
| --- | --- | --- | --- |
| B1 | Number of pregnancies | …………… |  |
| B2 | How many children do you have? | …………… |  |
| B3 | Did you seek antenatal care during the last pregnancy? | Yes | 1 |
|  |  | No | 2 |
| B4 | If yes, how many times did you receive antenatal care? | …………….. |  |
| B5 | When did you receive antenatal care? | ……………… |  |
| B6 | Did you experience any complications in your previous pregnancies? | Yes | 1 |
|  |  | No | 2 |
| B7 | If yes, what type of complications were identified? | Pre-eclampsia | 1 |
|  |  | Eclampsia | 2 |
|  |  | High Blood Pressure | 3 |
|  |  | Gestational Diabetes | 4 |
|  |  | Malpresentation | 5 |
|  |  | Rh-Negative | 6 |
|  |  | Vaginal bleeding | 7 |
|  |  | Ectopic Pregnancy | 8 |
|  |  | Prolonged/obstructed labour | 9 |
|  |  | Foetal distress | 10 |
|  |  | Placental Complication | 11 |
|  |  | IUGR | 12 |
|  |  | Other (specify)……...... | 13 |
| B8 | In which health facility did you give birth? | Health post/ Birthing Center | 1 |
|  |  | Primary Health Care Center | 2 |
|  |  | Government Hospital | 3 |
|  |  | Private Hospital/ Clinics | 4 |
|  |  | Other (specify)……...... | 5 |
| B9 | If secondary/ tertiary hospital, were you referred by the primary health facility? | Yes |  |
|  |  | No |  |
| B10 | Did you experience any complications in your last pregnancy? | Yes | 1 |
|  |  | No | 2 |
| B11 | If yes, what type of complications did you experienced in your last pregnancy? | Pre-eclampsia | 1 |
|  |  | Eclampsia | 2 |
|  |  | High Blood Pressure | 3 |
|  |  | Gestational Diabetes | 4 |
|  |  | Malpresentation | 5 |
|  |  | Vaginal bleeding | 6 |
|  |  | Prolonged/obstructed labour | 7 |
|  |  | Foetal distress | 8 |
|  |  | IUGR | 9 |
|  |  | Retained Placenta | 10 |
|  |  | Ruptured uterus | 11 |
|  |  | Previous CS | 12 |
|  |  | Other (specify)…………..….. | 13 |
| B12 | How far is the health facility you visited for your last childbirth from your residence? | ……hour …… minutes |  |
| B13 | What is your usual mode of transportation to reach the health facility? | Walking | 1 |
|  |  | Bike | 2 |
|  |  | Car | 3 |
|  |  | Public Vehicle | 4 |
|  |  | Taxi | 5 |
|  |  | Other (specify)…….. | 6 |
| B14 | Which health facility is closest to your residence? | Health post/ Birthing Center | 1 |
|  |  | Primary health care Center | 2 |
|  |  | Government Hospital | 3 |
|  |  | Private Hospital | 4 |
|  |  | Other (specify)…….. | 5 |
| B15 | Name the Health Facility | ….......................... |  |
| B16 | How far is the nearest birthing facility from  your residence? | ……hour …… minutes |  |
| B17 | Have you ever visited your nearest birthing facility? | Yes |  |
|  |  | No |  |
| B18 | Who influence you to go to the health facility for delivery? | Self | 1 |
|  |  | Husband | 2 |
|  |  | In-laws | 3 |
|  |  | FCHV | 4 |
|  |  | Neighbor | 5 |
|  |  | Health Workers | 6 |
|  |  | Other (specify)…….. | 7 |
| B19 | Are you involved in mother's  group? | Yes | 1 |
|  |  | No | 2 |
| B20 | Was there any discussion held regarding the place of delivery in mother's group? | Yes | 1 |
|  |  | No | 2 |
| B21 | Are you involved in income saving group? | Yes | 1 |
|  |  | No | 2 |
| B22 | Are you involved in social works? | Yes | 1 |
|  |  | No | 2 |

|  | **Satement** | **Disagree (1)** | **Neutral (2)** | **Agree (3)** |
| --- | --- | --- | --- | --- |
| **C1** | **Quality of the nearest birthing center** | | | |
| C1.1 | In your opinion, the number of health staff in the health facility is adequate. |  |  |  |
| C1.2 | In your opinion, the health staffs in the health facility are well suited to treat provide delivery services. |  |  |  |
| C1.3 | In your opinion, the laboratory services provided by the health facility is adequate. |  |  |  |
| C1.4 | In your opinion, patients can obtain drugs from this health facility easily. |  |  |  |
| C1.5 | In your opinion, the equipment in the health facility is well suited for delivery services |  |  |  |
| C1.6 | In your opinion, the provision of clean drinking water, hand washing facilities, and toilets for women in the facility are adequate. |  |  |  |
| C1.7 | In your opinion, the overall environment of the health facility is very clean. |  |  |  |
| C1.8 | In your opinion, there is separate room and maintains privacy during check-up in the health facility. |  |  |  |
| C1.9 | In your opinion, the health facility provides timely ambulance service. |  |  |  |
| C1.10 | In your opinion, the opening time of the health facility is adequate. |  |  |  |
| C1.11 | In your opinion, the waiting time in the health facility is adequate. |  |  |  |
| **C2** | **Competences of Health workers of the nearest birthing center** | | | |
| C2.1 | In your opinion, the time that the health workers devote to their patients is adequate. |  |  |  |
| C2.2 | In your opinion, the health workers are respectful towards the patients |  |  |  |
| C2.3 | In your opinion, the health workers at the nearest health facility are capable of handling birth related complications |  |  |  |
| C2.4 | In your opinion, the health workers at the nearest health facility provide full information about your health |  |  |  |
